# EPCGen2 Pseudorandom Number Generators: Analysis of J3Gen

**DOI:** 10.3390/s140406500

**Published:** 2014-04-09

**Authors:** Alberto Peinado, Jorge Munilla, Amparo Fúster-Sabater

**Affiliations:** 1 Escuela Técnica Superior de Ingeniería de Telecomunicación, Universidad de Málaga, Málaga, Spain; E-Mail: apeinado@ic.uma.es; 2 Instituto de Tecnologías Físicas y de la Información, Consejo Superior de Investigaciones Científicas, Madrid, Spain; E-Mail: amparo@iec.csic.es

**Keywords:** pseudo random number generators, security, cryptanalytic attack, Radio Frequency Identification, EPCglobal Gen2

## Abstract

This paper analyzes the cryptographic security of J3Gen, a promising pseudo random number generator for low-cost passive Radio Frequency Identification (RFID) tags. Although J3Gen has been shown to fulfill the randomness criteria set by the EPCglobal Gen2 standard and is intended for security applications, we describe here two cryptanalytic attacks that question its security claims: (i) a probabilistic attack based on solving linear equation systems; and (ii) a deterministic attack based on the decimation of the output sequence. Numerical results, supported by simulations, show that for the specific recommended values of the configurable parameters, a low number of intercepted output bits are enough to break J3Gen. We then make some recommendations that address these issues.

## 1. Introduction

The term “Internet of Things” (IoT) was coined in 1999 by Kevin Ashton, a co-founder of the Auto-ID Center [1], a center that promoted the research and development of tracking products for supply-chains by using low-cost Radio Frequency Identification (RFID) tags. The advantages of RFID over barcode technology are that it is wireless, does not require direct line-of-sight and that tags can be interrogated at greater distances, faster and concurrently [2]. This makes the IoT a wireless network of objects and sensors that collect and process information autonomously. RFID tags and sensors enable computers to observe, identify and understand for situational awareness without the limitations of human-entered data.

Nowadays, RFID is already a mature technology, and it is widely deployed for supply-chain, retail operations, inventory management and automatic identification in general. A typical RFID architecture involves three main components: (i) tags or transponders, which are electronic data storage devices that are attached to the objects to be identified; (ii) readers or interrogators, which manage the tag population, read data from and write data to tags; and (iii) a back-end server, which is a trusted entity that exchanges tag information with the readers and processes these data according to the specific intended application. Most tags are passive, which means that they do not have any kind of battery and receive the energy that they need to work from the reader. Thus, tags are inactive till they pass through the electromagnetic field generated by a reader, which is tuned to the same frequency.

The initial designs of RFID protocols focused on the performance with little attention being paid to resilience and security. However, as early as 2002, the first papers pointing out some possible security and privacy issues were already published, and in 2003, the CASPIAN (Consumer Against Supermarket Privacy Invasion and Numbering) group raised concerns regarding the possible misuse of RFID technology and called for boycotts against companies that decided to incorporate them. The European Commission in 2008 launched a public consultation on the issues of the use of RFID technology, particularly in terms of privacy, data protection and information security [3]. RFID can be indeed used to perform different forms of privacy invasion, such as unauthorized reading or tracking of people, and can be subject to impersonation. To overcome these issues, apart from the legal pressure for the protection of personal information (e.g., [4] in Europe and [5] in the U.S.), the technical means to control the access to tags is the implementation of cryptographic mechanisms that take into account their special characteristics: power-constrained devices, the vulnerability of the radio channel, reply upon-request, *etc*.

This increasing concern about security is evidenced with the inclusion of some optional cryptographic features in the recently ratified (November 2013) second version of the EPCglobal Gen2 Specification [6]. EPCglobal Gen2, hereafter EPCG2, is the standard (ISO [7]) for low-cost tags that work in the Ultra High Frequency (UHF) band of 860–960 MHz. This defines a platform for RFID protocol interoperability and supports basic reliability guarantees, provided by a 16-bit cyclic redundancy code (CRC16) and an on-chip 16-bit pseudo-random number generator (PRNG). The first version was published in 2004, and since then, there have been many attempts to secure EPCG2 protocols with the use of the passwords defined by the standard (e.g., [8,9]) or based on the CRC (e.g., [10,11]). Nevertheless, practically, they all have proven unsuccessful, due to the length of the keys, which are also static, and the linearity properties of CRC [12]. As a result, PRNG has become the key element in most security protocols proposed in the literature for this kind of tag (e.g., [13–17]). These protocols are based on the assumption that the PRNG implemented in the tag is cryptographically secure. In the new version (second) of the standard, tags may support one or more cryptographic suites (which must be specified), but then again, these would most likely require the implementation of a secure PRNG. The PRNG is also used for some processes, such as the anti-collision algorithm or link-cover coding (a basic privacy mechanism described in the standard). Nevertheless, despite its practical significance, EPCG2 does not specify any possible PRNG implementation, and although security through obscurity has shown to be not advisable (e.g., [18,19]), manufacturers are still reluctant to make their designs publicly accessible. In addition, in the literature, there are only a few descriptions of PRNGs for low-cost RFID tags (e.g., [20–24]). Thus, as far as we know, the works of Melià *et al.* [25] and Mandal *et al.* [26], which is a modification of the previous one, are hitherto the only references that propose EPCG2 compliant PRNGs and that check how it meets the specific randomness requirements established by the standard.

Melià-Seguí *et al.* describe, in the first version [25] and then with more details in this journal [27], a PRNG for low-cost passive RFID tags (including, but not limited to EPCG2), called J3Gen, which provides a very high level of unpredictability, with a reduced computational complexity and low-power consumption. J3Gen is based on a linear feedback shift register (LFSR) configured with multiple feedback polynomials, and its authors claim that it is suitable for security purposes (e.g., [28,29]).

Nevertheless, in this paper we analyze the design of J3Gen and show that the security level provided by this PRNG falls well short of its security claims. Two different cases, for two different sets of suggested parameters, are analyzed. As a result, the randomness of the generated sequences decreases dramatically, and its use for security applications is questioned. We then suggest some values for the choice of parameters that could hinder these cryptanalyses, as well as some possible changes to strengthen the protocol. Note that the security of many cryptographic systems depends upon the generation of pseudo random numbers, and therefore, the weaknesses of the PRNG impact on the security of the global systems and may be exploited to attack them; e.g., the attack on Crypto-1, a cryptosystem for use on MIFARE chips [30].

The rest of the paper is organized as follows. Section 2 introduces some general concepts about EPCG2 PRNGs and describes the structure and the characteristics of J3Gen in particular. Section 3 describes the cryptanalysis of J3Gen for two different sets of recommended parameters. Then, in Section 4, we comment on some possible modifications to improve J3Gen, and finally, Section 5 concludes the paper.

## 2. An EPCG2-Compliant PRNG: J3Gen

**Definition 1 (PRNG)**. *A PRNG is a pseudo-random bit generator (PRG) whose output is partitioned into blocks of a given length, n. Each block defines an n-bit number, said to be drawn from the PRNG*.

**Definition 2 (PRG)**. *A PRG is a deterministic algorithm that, on inputting a binary string of length K, called the seed, generates a binary sequence, s, of length S* >> *K which “appears” to be random.*

While it is very difficult to give a mathematical proof that a PRNG is indeed secure, we gain confidence by subjecting it to a variety of statistical tests designed to detect the specific characteristics expected of random sequences (we refer the reader to [31] for a comprehensive collection of randomness tests). Although the new version of EPCG2 explains that the different implemented cryptographic suites may define more stringent requirements for the PRNG [6], these are the “basic” randomness criteria set by the standard:

1. **Probability of a single ***RN*16: The probability that any *RN*16 drawn from the PRNG has value *RN*16 = *j*, for any *j*, shall be bounded by:
  $$0.8/2^{16}<\mathrm{Prob}(RN16=j)<1.25/2^{16}$$
2. **Probability of simultaneously identical sequences:** For a tag population of up to 10,000 tags, the probability that any two or more tags simultaneously generate the same sequence of *RN*16*s* shall be less than 0.1%, regardless of when the tags are energized.
3. **Probability of predicting an ***RN*16: An *RN*16 drawn from a tag's PRNG shall not be predictable with a probability better than 0.025%, when the outcomes of prior draws from the PRNG under identical conditions are known.

According to the authors, J3Gen amply fulfills these requirements, providing a high level of security (an equivalent key size of 372 bits). We find, however, some flaws in the design of this PRNG that question the validity of this proposal. Before analyzing these issues, we describe the structure and the characteristics of J3Gen.

### 2.1. Description of J3Gen

J3Gen is based on a dynamic linear feedback shift register (DLFSR) of *n* cells. A DLFSR can be defined, in turn, as an LFSR [32] where the feedback polynomial, *p_i_*(*x*), is not static, but changes dynamically [33]. J3Gen combines this DLFSR topology with a physical source of true randomness (thermal), which generates a “true random bit”, denoted by *trn*. This bit controls the change of polynomials, preventing the linear behavior of the DLFSR. This *trn* is replaced by a PRNG in [26].

**Figure 1.**
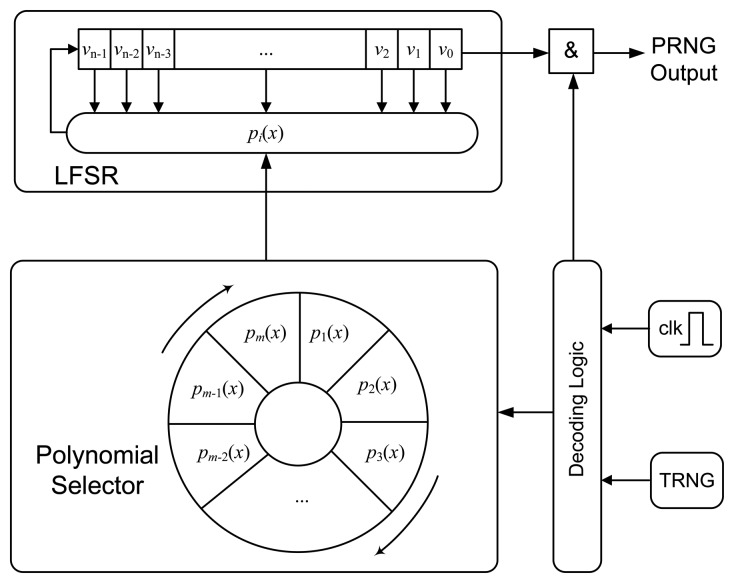
Block diagram of J3Gen. PRNG, pseudo-random number generator; LFSR, linear feedback shift register.

Figure 1 depicts the block diagram of J3Gen. A set of *m* primitive feedback polynomials are implemented as a wheel, and the polynomial selector rotates one position if *trn* = 0 and two positions (one position at one shift cycle and another at the next shift cycle) if *trn* = 1. These rotations are performed every *l* cycles, with 1 ≤ *l* < *n*. This value must be lower than *n* (the number of cells) to prevent a random number from being generated by a single feedback polynomial. The decoding logic is responsible for managing the internal PRNG clock and the *trn* bit, providing the correct signal to the different internal modules.

For a better understanding of the functioning of J3Gen, we review here the sample of the execution provided in [25]. The parameter configuration chosen for this example is: *n* = 16, *m* = 8 and *l* = 15. The value *n* = 16 for the LFSR size is selected because of compatibility reasons with EPCG2 (although larger values of *n* are also considered in [27]). The selected feedback polynomials (*m* = 8) should remain secret, since they can be considered as the secret key of the system. In [25], the LFSR states for 32 shift cycles are detailed, providing 32 output bits. Two true random values are used, which are set to *trn*_1_ = 0 and *trn*_2_ = 1. The system starts with *p*_1_(*x*) and outputs 15 (= *l*) bits until the TRNGmodule transfers *trn*_1_ = 0 to the decoding logic module. Then, *p*_2_(*x*) is selected, and another 15 bits are generated, until the next (after *l* shift cycles) *trn* is obtained. As *trn*_2_ = 1, the decoding logic rotates the polynomial selector one position at Shift 31 and another position at Shift 32. Eventually, two 16-bit pseudorandom numbers are generated; for the first one, *p*_1_(*x*) is used 15 cycles and *p*_2_(*x*) one cycle, while for the second one, *p*_2_(*x*) is used 14 cycles and then *p*_3_(*x*) and *p*_4_(*x*) are used for one cycle each (*p*_4_ will also be used 14 cycles for the next 16-bit pseudorandom number).

### 2.2. Security Strength

In [27], the authors equate the security strength of J3Gen with a key length, where each possible key corresponds to a possible feedback polynomial combination (which must be kept secret). Thus, for *n* = 16 and *m* = 8, this would mean a key size of roughly 73 bits; *i.e.*, 8 (= *m*) selected feedback polynomials out of 2,048 possible primitive polynomials of degree 16 (= *n*). Apart from the parameters, *n* and *m*, the polynomial update cycle, *l*, has also a major impact on the security level. For example, with *n* = 32 and *m* = 16, for *l* = 31, the authors compute that there will be up to four possible solutions for each system of equations, *i.e.*, up to four possible feedback polynomials could be involved in the generation of such a sequence. If *l* = 25, then the possible solutions are up to 16,384; for *l* = 21, the possible solutions increase up to 4,194,304, and so on, until *l* = 1, the extreme case, where all 67 million primitive feedback polynomials would be equally probable.

### 3. Cryptanalysis of the J3Gen

This section analyzes the security of J3Gen, describing two procedures that enable: (i) retrieving of the feedback polynomials; and (ii) reconstructing the sequence. These cryptanalyses have been carried out for *l* = *n* − 1 and *l* = 1, respectively. The former is the value selected for the execution sample in [25], and the latter is pointed out as the most secure option by the designers [27].

#### 3.1. Case l = n − 1: Retrieving the Feedback Polynomials

This first analysis shows that the security evaluation carried out by the authors (see Section 2.2) presents some flaws. According to the given data, it can be inferred that the number of possible feedback polynomials involved in the generation of a 16-bit pseudorandom number is estimated to be 2^2(^*^n^*^−^*^l^*^)^. Nevertheless, we show here that this number can be reduced dramatically to just 2*^n^*^−^*^l^*. As a consequence, the case *l* = *n*−1 becomes particularly vulnerable, and the feedback polynomials can be retrieved. This problem gets much worse when the adversary makes use of some known characteristics of the feedback polynomials.

##### 3.1.1. Cryptanalysis Description

The knowledge of 2*n* output values of a sequence, *s*, generated with an *n*-degree feedback polynomial enables the definition of a linear equation system of *n* equations to retrieve such a polynomial:

(1) $$O_{n\times 1}=S_{n\times n}\cdot C_{n\times 1}\Rightarrow \left(\begin{array}{c} s_{n+1}\\ \vdots \\ s_{2n} \end{array}\right)=\left(\begin{array}{ccc} s_{n} & \cdots & s_{1}\\ \vdots & \ddots & \vdots \\ s_{2n-1} & \cdots & s_{n} \end{array}\right)\cdot \left(\begin{array}{c} C_{1}\\ \vdots \\ C_{n} \end{array}\right)$$

**Figure 2.**
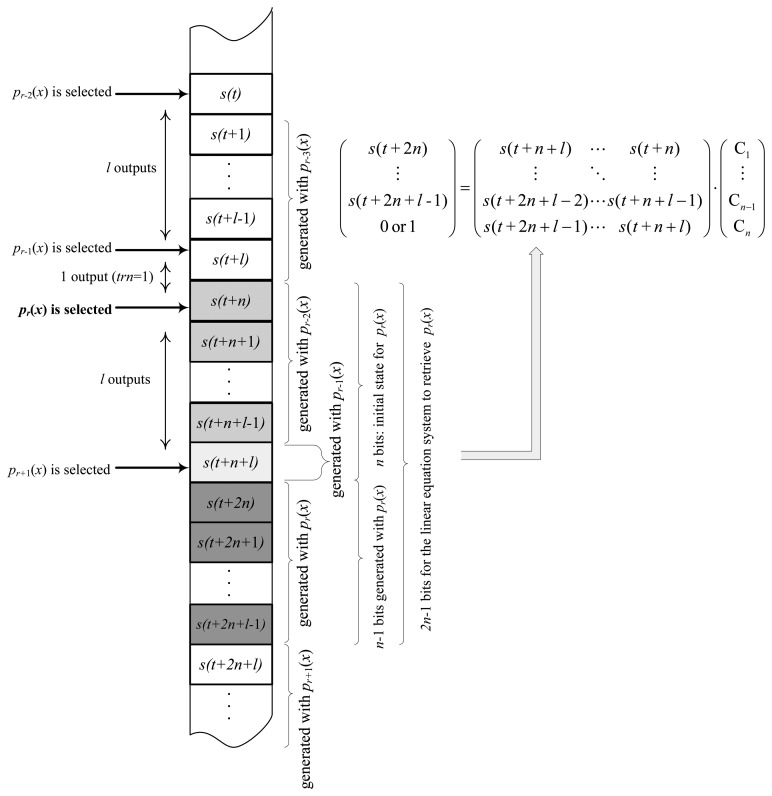
Sketch of the linear equation system definition.

J3Gen prevents us from obtaining these values by changing the feedback polynomial after *l* rounds, with *l* < *n.* However, analyzing how DLFSR-based PRNGs work, it can be noticed that for *l* = *n* − 1, there is only one unknown bit left to define this linear equation system. Thus, an adversary just needs to try with the two possibilities, *i.e.*, zero and one, and checks if there exists a valid solution (only two feedback polynomials are involved). Figure 2 sketches, for a polynomial, *p_r_*(*x*), how the corresponding matrices are defined. When a feedback polynomial is selected by the polynomial selector, its initial state is known, as it corresponds with the following *n* outputs. If this feedback polynomial is used now to generate *l* = *n* − 1 outputs, we will then know a total of 2*n* − 1 bits.

We have just shown that a feedback polynomial, *p_r_*(*x*), could be retrieved from 2*n* − 1 outputs of J3Gen. However, if these 2*n* − 1 bits are taken at random, with a high probability, fewer than *n* − 1 bits will have been generated with the same feedback polynomials, and the system will have no solution or this will be wrong. To overcome this problem, if more outputs of J3Gen are available, the adversary only has to shift one position after another and test if the defined system has a valid solution. If so, the polynomial is stored as a candidate. A maximum of *n* (if *trn* = 1) shifts will be needed before finding a correct solution. Finally, the adversary will have to pick up the correct feedback polynomials among the possible candidates. For this last task, different alternatives are possible, which will be commented on with an example in the next subsection. Given a J3Gen generated sequence, *s*, of length *S* ≥ *n*+*l* bits and letting *s_k_* be the *k*-th bit of *s*, Algorithm 1 collects more formally the different steps of this cryptanalysis.

This general procedure can be further optimized when certain information about the feedback polynomials is known. For example, in J3Gen, the feedback polynomials are primitive. We know then that the number of non-zero terms is odd, with *C*_0_ = *C_n_* = 1. Thus, *n* − 2 equations, derived from just 2*n* − 2 outputs, can be used to recover coefficients *C_i_* with *i* ∈ [1:(*n* − 2)], while *C_n_*_−1_ will be the odd parity bit of such coefficients. This way, a submatrix *R* = *S*_(_*_n_*_−2)×(_*_n_*_−2)_ with the first (*n* − 2) rows and columns of the previously defined *S_n_*_×_*_n_* can be used to solve the linear equation system, while the (*n* − 1)-th and *n*-th rows of this matrix represent two spare equations that will only be included when required; that is, (i) when rank(*R*) = rank(*R* | *O*) = *n* − 4 or (ii) when rank(*R*) = rank(*R* | *O*) = *n* − 3. In the first case, we need to include both equations ((*n* − 1) and *n*), while in the second case, we include firstly the (*n* − 1)-th row, and only if the rank does not change (*i.e.*,rank(*R*) = rank(*R* | *O*) = *n* − 3); the *n*-th row is included.



**Algorithm 1** Procedure to retrieve the J3Gen feedback polynomials.
 Given a sequence, *s*, of length *S* ≥; *n*+*l*  for *i* = 1 to (*S* − (*n* + *l*) + 1)   Take *n* + *l* bits of *s: h* = *s_i_*…*s_i_*_+_*_n_*_+_*_l_*_−1_   Set *h*_2_*_n_* = *j* with *j* = 0,1 and solve the linear equation systems.    If there exist a valid solution, then store it as a candidate.  end  Select the feedback polynomials among the candidates.


##### 3.1.2. Cryptanalysis of the Given J3Gen Example

**Figure 3.**
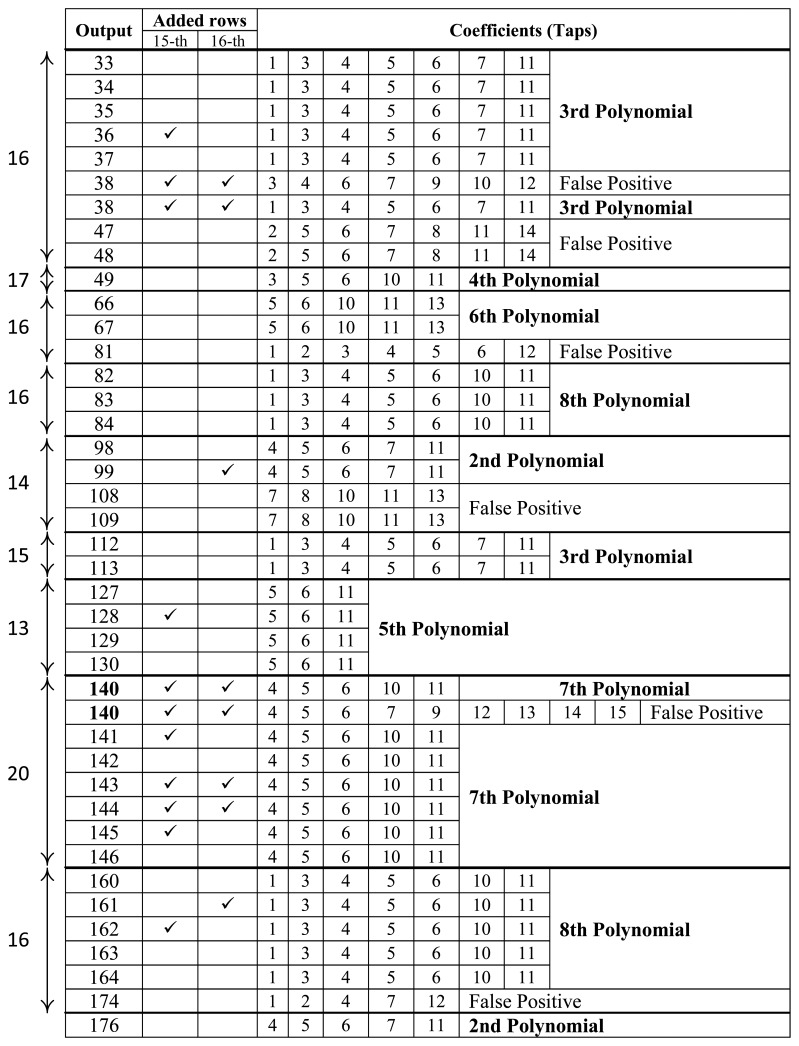
Example of the results of the cryptanalysis.

To illustrate the cryptanalysis, we apply it on the example described above (Section 2.1): *n* = 16, *m* = 8, *l* = 15 and the primitive feedback polynomials of Table 1. Figure 3 shows the results of the cryptanalysis on a bitstring, *s*, of length *S* = 176 bits. There are 14 different candidates; seven of them correspond with actual used feedback polynomials, while the other six are “false positives”. It is easy to note, however, that the frequency of appearance of genuine polynomials is much higher than that of “false positives”; when a candidate is found for a specific output, the probability of this being genuine is roughly 75%. Table 2 shows the average number of correct polynomials between the candidates for different lengths, *S*, and the number of them that are correct among the most frequent ones (chosen candidates). These results are obtained with the Monte-Carlo method for 1,000 repetitions. They show that 128 bits are enough to recover five of the eight feedback polynomials. These results can still be improved if the method to discard the “false positives” (*i.e.*, to choose among the candidates) is refined. For example, one could take into account the output when the candidate is obtained, as genuine solutions appear roughly every *n* outputs and/or if two solutions are found for the same output (Output 140 in Figure 3). In this case, only one of them can be correct, and therefore, the other can be ruled out directly.

Finally, note that this cryptanalysis does not require that the given outputs are consecutive. An adversary, with several outputs of length *S*^1^, *S*^2^,…*S^n^*, can perform *n* independent analyses to retrieve one or several polynomials with each of them (provided that *S^i^* ≥ *l* + *n*).

##### 3.1.3. Discussion for Different Values of the Parameters

The cryptanalysis described above can be applied for any value of *m*. It only affects the number of outputs, *S*, that the cryptanalysis needs to recover all of the polynomials; (*n* + *m* · *l*) bits could be enough to recover up to *m* feedback polynomials. Each polynomial that is retrieved reduces the equivalent key size.

**Figure 4.**
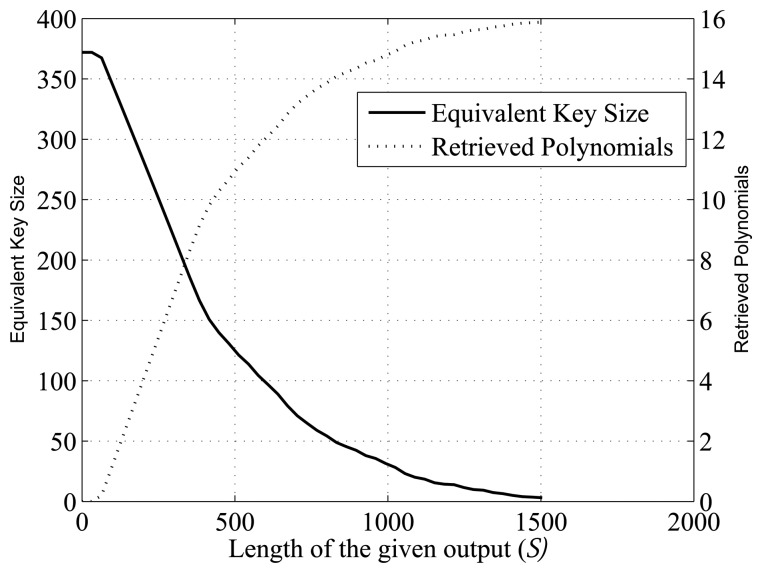
Results of the cryptanalysis for *m* = 16, *n* = 32 and *l* = 31.

There is also no significant changes in the cryptanalysis when *n* > 16, other than the size of the linear equation systems and the number of shifts to define these. In [27], the authors opt for *m* = 16 and *n* = 32 as the best implementation in terms of security and hardware complexity. This configuration is supposed to provide an equivalent key of 372 bits (16 out of 67,108,864 primitive polynomials of degree 32). Nevertheless, 528 output bits could be enough to recover all of the 16 feedback polynomials, and we find that an adversary with an output sequence of 1,152 bits is able to recover all of these polynomials in half of the cases. Figure 4, based on simulations and statistical analysis, shows an approximation of the average number of polynomials that are retrieved for different lengths of the given outputs and how the equivalent key size reduces when these polynomials are disclosed.

The cases *l* = *n* − 2 and *l* = *n* − 3 with primitive feedback polynomials are essentially a particular case of the analysis described (with one or none of the spare equations). Apart from that, this cryptanalysis could be also applied to different values of *l* with a complexity significantly lower than that estimated by the authors [27]: 2*^n^*^−^*^l^* possibilities instead of 2^2(^*^n^*^−^*^l^*^)^. However, this still leads to an ever-increasing computational complexity when *n* − *l* increases, as the number of possibilities also does. Therefore, in this case, we suggest to consider other strategies. In the next section, for example, we describe a different approach to break J3Gen when *n* − *l* is maximum (*l* = 1).

#### 3.2. Case l = 1: Reconstructing the Output Sequence

This section analyzes the J3Gen outputs for the case when *l* = 1, which is suggested as the most secure choice. This cryptanalysis is, as mentioned, different from the previous one and even more powerful. While the results in the previous case were probabilistic, we show here that the generated sequence can be reconstructed in a deterministic way. The analysis exploits a design fault in J3Gen that renders useless the randomness provided by *trn*; when *l* = 1, the *m* feedback polynomials are applied consecutively, *i.e.*, [*p*_1_(*x*), *p*_2_(*x*),…, *p_m_*(*x*), *p*_1_(*x*), *p*_2_(*x*),…]. Indeed, if *trn* = 0, the following output bit is computed by using the next feedback polynomial, *p_i_*_+1_(*x*), and then, another *trn* is obtained. If *trn* = 1, the system generates two output bits, by using *p_i_*_+1_(*x*) and *p_i_*_+2_(*x*), respectively, and then, another bit *trn* is drawn. Thus, the true random bit *trn* does no provide any randomness to the process, and each feedback polynomial will be periodically applied with period *m*. This fact makes it possible to apply a cryptanalysis, such as that applied to programmable cellular automata (PCA) [34] and DLFSR [33], considering the output sequence, *s*, as an interleaved sequence composed by decimated sequences (*cf*. [35] for the theory on interleaved sequences).

##### 3.2.1. Cryptanalysis Description

Let *s* be the output binary sequence produced by the J3Gen generator where the generic term *s*(*t*) = *v*_0_(*t*) is the least significant bit of the state *v*(*t*) = (*v_n_*_−1_(*t*), …,*v*_1_(*t*), *v*_0_(*t*)) of the LFSR at time instant *t*. The linear span of this random sequence can be defined as follows:

**Definition 3** (Linear Span). *The linear span (or linear complexity, notated as LC) of a binary sequence, s, is defined as the length of the shortest LFSR that can generate such a binary sequence.*

Let us also define the sequence, *w*_0_, as a decimation of the sequence, *s*, by taking one term out of *m*; that is,

(2) $$w_{0}\left(t\right)=s\left(t\cdot m\right)\mathrm{for}t\geq 0$$

The following equation holds between the states of the LFSR,

(3) v((t+1)⋅m)=v(t⋅m)]M

where:

(4) $$M=\overset{i=m}{\underset{i=1}{\Pi }}A_{i}$$

*A_i_* being a *n*×*n* matrix, whose characteristic polynomial is the feedback polynomial, *p_i_*(*x*), of the LFSR. Thus, it can be written that:

(5) $$w_{0}\left(t\right)=s\left(t\cdot m\right)=\pi \left(M^{t}\cdot v\left(0\right)\right)$$

where *π* is a linear map of an *n*-dimensional vector space over *GF*(2) that transforms (*v_n_*_−1_(*t*),…, *v*_1_(*t*), *v*_0_ (*t*)) into *v*_0_(*t*).

The term, *w*_0_(*t* + *n*), can be written [33,34] as a linear combination of the previous *n* terms, and consequently, the linear span of the sequence, *w*_0_, is at most *n*. The same reasoning applies to any of the other decimated sequences, *w_j_*, whose generic terms are defined as:

(6) $$\begin{array}{cc} w_{j}\left(t\right)=s\left(t\cdot m+j\right), & 0\leq j\leq \left(m-1\right) \end{array}$$

This way, the sequence, *s*, can be obtained by interleaving *m* different sequences *w_j_*, where each of them has a linear span *LC* ≤ *n*. Thus, the linear span of *s* is upper bounded as *LC*(*s*) ≤ *n* · *m*, which means that the sequence, *s*, can be reconstructed from the knowledge of at most 2*n* · *m* bits. Consequently, for *n* = 16 and *m* = 8, the binary sequence produced by J3Gen can be reconstructed from just 256 consecutive bits (or 16 pseudorandom numbers), using an equivalent LFSR of 128 stages. In the case of *n* = 32 and *m* = 16, the output sequence can be rebuilt by using 1,024 consecutive bits (or 64 pseudorandom numbers). Note that such sequences can be reconstructed without the knowledge of the feedback polynomials.

**Figure 5.**
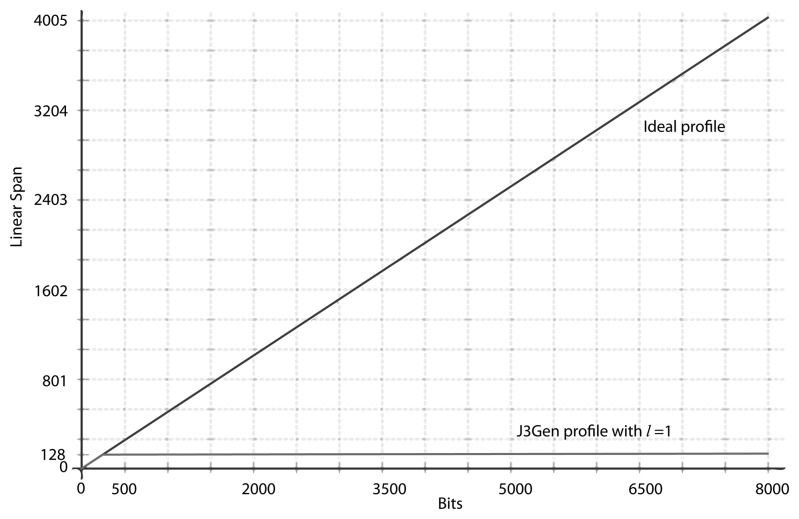
The linear span profile of the first 8,000 bits in a sequence of a length of 30,016 bits produced by the J3Gen PRNG with *n* = 16, *m* = 8 and *l* = 15.

**Figure 6.**
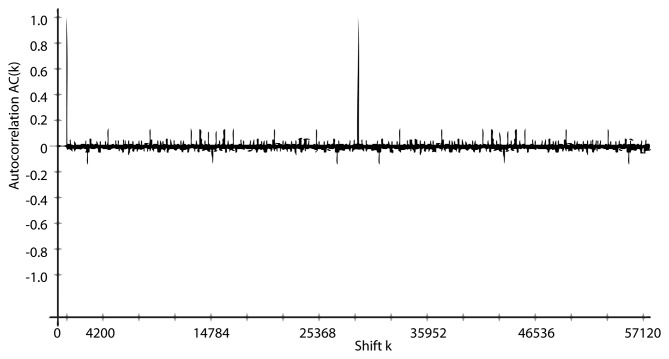
Autocorrelation of a sequence generated with *n* = 16, *m* = 8 and *l* = 15.

These conclusions are confirmed when the Massey-Berlekamp algorithm [36] is applied on sequences generated by J3Gen with *n* = 16, *m* = 8 and *l* = 1 (the feedback polynomials of Table 1). The results reveals a linear span *LC* = 128 with an equivalent feedback polynomial *p_eq_*(*x*) = *x*^128^ + *x*^120^ + *x*^88^ + *x*^80^ + *x*^72^ + *x*^56^ + 1, whose order, which determines the period of the sequence, is 28, 560. Figure 5 and Figure 6 depict the linear span profile and the repetition period, respectively.

### 4. Security Recommendations for J3Gen

In [27], the authors suggest that the pool of feedback polynomials could include non-primitive polynomials to increase the number of possible combinations and, thus, prevent J3Gen from a brute force attack. This certainly would hinder the cryptanalysis described in Section 3.1, as the process to discard candidates (the last step in Algorithm 1) would become harder. However, this modification needs to be done carefully, since non-primitive polynomials produce sequences whose statistical properties are not guaranteed (must be proven). Furthermore, the selection of these feedback polynomials should not apply any fixed rule that could leak information about the selected protocols. For example, the polynomials in Table 1 seem to apply that used in similar architectures (e.g., [37]), which looks for an efficient hardware implementation by choosing polynomials with several coefficients in common: all polynomials share coefficients *x*^16^, *x*^11^, *x*^6^, *x*^5^ and *x*^0^. This simplifies the logic circuitry (fewer gates) to select the appropriated polynomials from the pool, but obviously impacts negatively on the global security of the PRNG.

Regarding the analysis described in Section 3.2, an alternative way to obtain the period of the sequence is with the computation of the matrix, *M* (see Equation (4)). This matrix can also be computed from 2*n* · *m* consecutive bits taking only 2*n* bits of a decimated sequence (one out of *m*). The computation of *M* is equivalent to the computation of the equivalent LFSR determined by the linear span of a decimated sequence. Once the matrix is computed, its characteristic polynomial, *c*(*x*), gives us information about the period of the sequence [33]. This matrix, *M*, is completely determined by the feedback polynomials and the order in which they are applied. Thus, it is possible to know *a priori* the period of the output binary sequence computing the characteristic polynomial of the matrix, *M*. The polynomials proposed in [25] (listed in Table 1) determine a matrix, *M*, whose characteristic polynomial is: *c*(*x*) = *x*^16^ + *x*^9^ + *x*^7^ + *x*^6^ + *x*^5^ + *x* +1 = (*x*^8^ + *x*^7^ + *x*^5^ + *x*^3^ + 1)(*x*^2^ + *x* + 1)(*x*^3^ + *x* + 1)^2^. The order of this non-primitive polynomial is 3,570. Since *c*(*x*) determines the period of all decimated sequences [33,35], the period of the interleaved sequence, *s*, is 3,570 · *m* = 28, 560, a much lower value than the maximum length, 65,535, produced by a single primitive LFSR of a length of 16. However, a simple modification in the order of the polynomials may increase the repetition period of the sequence. For example, if *p*_1_(*x*) and *p*_5_(*x*) interchange their positions, then the repetition period is maximized; the characteristic polynomial of the matrix, *M*, is *c*(*x*) = *x*^16^ + *x*^14^ + *x*^13^ + *x*^12^ + *x*^10^ + *x*^8^ + *x*^7^ + *x* + 1, which is primitive and determines a period of 65, 536 for the decimated sequences and 65, 536 · 8 = 524,288 for the interleaved sequence. However, the pool of polynomials cannot significantly improve the upper bound of the linear span, since it does not depend on the specific implemented polynomials, but on their number and degree; *LC* ≤ *n*·*m* (Section 3.2). Furthermore, the linear span will always be too low compared to the repetition period. As a consequence, we strongly advise against the use of *l* = 1.

### 5. Conclusions

In the present work, we have analyzed the security of J3Gen. J3Gen is one of the few PRNGs described in the literature that is suitable to be implemented in low-cost RFID tags and the only one, as far as we know, that has been shown to fulfill the randomness criteria established by the EPCglobal Gen2 standard. Nevertheless, despite its security claims, we have described here two distinct cryptanalytic attacks for the two values of the parameter, *l*, recommended by the designers:

- *l* = *n*−1: A probabilistic cryptanalysis based on solving linear equation systems is introduced. This analysis allows one to recover the set of feedback polynomials, which constitute the secret information of J3Gen. No more than (*n* + *m* · *l*) output bits could be enough to accomplish this task. In addition, cases *l* = *n* − 2 and *l* = *n* − 3 are essentially straight derivations of the same analysis when the feedback polynomials are primitive.
- *l* = 1: A deterministic cryptanalysis based on the output sequence decimation is developed. This analysis shows that the entire output sequence of J3Gn can be reconstructed by the knowledge of 2 *nm* bits of such a sequence.

Although these analyses undoubtedly question the security of J3Gen, we enumerated some recommendations that address these issues and could be helpful for this or future designs.

## Figures and Tables

**Table 1.** Primitive feedback polynomials.

*p*_1_(*x*) : 1 + *x* + *x*^5^ + *x*^6^ + *x*^7^ + *x*^11^ + *x*^16^
*p*_2_(*x*) : 1 + *x*^4^ + *x*^5^ + *x*^6^ + *x*^7^ + *x*^11^ + *x*^16^
*p*_3_(*x*) : 1 + *x* + *x*^3^ + *x*^4^ + *x*^5^ + *x*^6^ + *x*^7^ + *x*^11^ + *x*^16^
*p*_4_(*x*) : 1 + *x*^3^ + *x*^5^ + *x*^6^ + *x*^10^ + *x*^11^ + *x*^16^
*p*_5_(*x*) : 1 + *x*^5^ + *x*^6^ + *x*^11^ + *x*^16^
*p*_6_(*x*) : 1 + *x*^5^ + *x*^6^ + *x*^10^ + *x*^11^ + *x*^13^ + *x*^16^
*p*_7_(*x*) : 1 + *x*^4^ + *x*^5^ + *x*^6^ + *x*^10^ + *x*^11^ + *x*^16^
*p*_8_(*x*) : 1 + *x* + *x*^3^ + *x*^4^ + *x*^5^ + *x*^6^ + *x*^10^ + *x*^11^ + *x*^16^

**Table 2.** Average retrieved polynomials.

***S***	**Correct Polynomials among the Candidates**	***v/w* = *v* Correct Polynomials among the *w* Most Frequent ones**
31	0.2	0.2 / 1
32	0.23	0.2 / 1
48	1.3	1.2 / 2
64	2.4	2.1 / 3
80	3.4	3.2 / 4
96	4.4	3.7 / 5
112	5.3	4.6 / 6
128	5.7	5 /7
144	6	5.5 / 8
160	5.9	5.4 / 8
176	6.7	6 /8
192	7.2	6.5 / 8
208	7.5	6.5 / 8
224	7.5	6.6 / 8
240	7.6	6.6 / 8
